# Air pollution and mortality for cancer of the respiratory system in Italy: an explainable artificial intelligence approach

**DOI:** 10.3389/fpubh.2024.1344865

**Published:** 2024-05-07

**Authors:** Donato Romano, Pierfrancesco Novielli, Roberto Cilli, Nicola Amoroso, Alfonso Monaco, Roberto Bellotti, Sabina Tangaro

**Affiliations:** ^1^Dipartimento di Scienze del Suolo, della Pianta e degli Alimenti Universita' degli Studi di Bari Aldo Moro, Bari, Italy; ^2^Istituto Nazionale di Fisica Nucleare Sezione di Bari, Bari, Italy; ^3^Dipartimento Interateneo di Fisica, “M. Merlin” Universita' degli Studi di Bari Aldo Moro, Bari, Italy; ^4^Dipartimento di Farmacia Scienze, del Farmaco Universita' degli Studi di Bari Aldo Moro, Bari, Italy

**Keywords:** explainable artificial intelligence, air pollution, lung cancer, respiratory disease, socio-economic indices, public health, remote sensing 2010 MSC: 00-01, 99-00

## Abstract

Respiratory system cancer, encompassing lung, trachea and bronchus cancer, constitute a substantial and evolving public health challenge. Since pollution plays a prominent cause in the development of this disease, identifying which substances are most harmful is fundamental for implementing policies aimed at reducing exposure to these substances. We propose an approach based on explainable artificial intelligence (XAI) based on remote sensing data to identify the factors that most influence the prediction of the standard mortality ratio (SMR) for respiratory system cancer in the Italian provinces using environment and socio-economic data. First of all, we identified 10 clusters of provinces through the study of the SMR variogram. Then, a Random Forest regressor is used for learning a compact representation of data. Finally, we used XAI to identify which features were most important in predicting SMR values. Our machine learning analysis shows that NO, income and O3 are the first three relevant features for the mortality of this type of cancer, and provides a guideline on intervention priorities in reducing risk factors.

## 1 Introduction

This study aims to investigate the relationship between air pollution and respiratory system cancer mortality in Italian provinces. Air pollution is a pressing issue of the modern world, impacting human health and the environment in numerous ways (1). One of its significant consequences is its link to respiratory system cancer, a malignancy that claims millions of lives globally each year (2). Air pollution, which mainly consists of fine particulate matter and toxic gases, can enter the lung tissue and trigger a series of chronic inflammatory and oxidative damage to the cells, leading to malignant transformation.

Respiratory system cancer is a type of cancer that affects the lungs, bronchi and trachea. The most common types of respiratory system cancer are lung cancer and bronchial cancer. (3). While smoking is a primary risk factor, there are other important contributing factors, such as secondhand smoke, occupational exposure, and air pollution. This sets the stage for the focus of the study (3). It is estimated that 14% of lung cancer deaths are attributable to environmental air pollution (4). Symptoms of respiratory system cancer can include persistent cough, chest pain, shortness of breath, hoarseness, fatigue, and weight loss.

A number of works have dealt with long and short-term effect of air pollution and cancer (5, 6), highlighting a multitude of contributing factors such as the type of pollutant, exposure time and frequency, and individual susceptibility (7): an increase of 10 μ*g/m*3 in PM10 concentration raises the average likelihood of developing lung cancer by 20%, whereas a 5 μ*g/m*3 rise in PM2.5 elevates the risk by 30% (8). Recognizing and addressing the link between air pollution and respiratory system cancer is critical for protecting public health on a global scale. It informs evidence-based policies, encourages international collaboration, and empowers individuals and communities to take actions that can mitigate the impact of air pollution on respiratory health.

The goal of this work was to implement a Machine Learning (ML) algorithm predicting the standard mortality ratio (SMR) for lung, bronchi and trachea cancer of the Italian provinces by using air pollution data downloaded from Copernicus Atmosphere Monitoring Service (CAMS) and socio-economic data downloaded from ISTAT. ML is the discipline dealing with the replication of the learning mechanisms of the human brain through statistical algorithms (9). Random forest is a machine learning algorithm used for both classification and regression tasks. It is an ensemble learning method that creates several decision trees and combines their predictions to make a final decision or prediction. Recent advancements in ML techniques resulted in the introduction of eXplainable Artificial Intelligence (XAI) which allows for the identification of the crucial attributes for each instance (10–12). Explainable AI provides clarity and understanding into the decision-making processes of AI models. It helps improve transparency, trust, accountability, and compliance with regulations. XAI methods has been applied to ML algorithms to provide a clear picture of the relevant features affecting the performance of the models, their relations with the outcomes, with each other's and both their local and global effects. The main intent of our study was to present a framework to determine which pollution indices, based on remote sensing data, are most associated to the mortality from cancer of the respiratory system. We studied the mortality in the Italian provinces given its heterogeneity in terms of ecological and environmental features. In order to do this, we evaluated to what extent mortality from cancer of the respiratory system can be predicted based on environmental pollution and socio-economic indices.

## 2 Materials

The study was conducted using mortality data from ISTAT, that is the Italian Institute of Statistics, responsible for collecting, analyzing, and disseminating official statistics on the country's population, economy, and society.^1^ ISTAT's main functions include conducting population censuses, compiling and publishing official statistics on topics such as, employment, and economic indicators, and providing support and expertise to other public institutions and organizations in the field of statistics. In particular, the respiratory system cancer mortality at the provinces level in 2019 has been considered.

**Figure 1:**
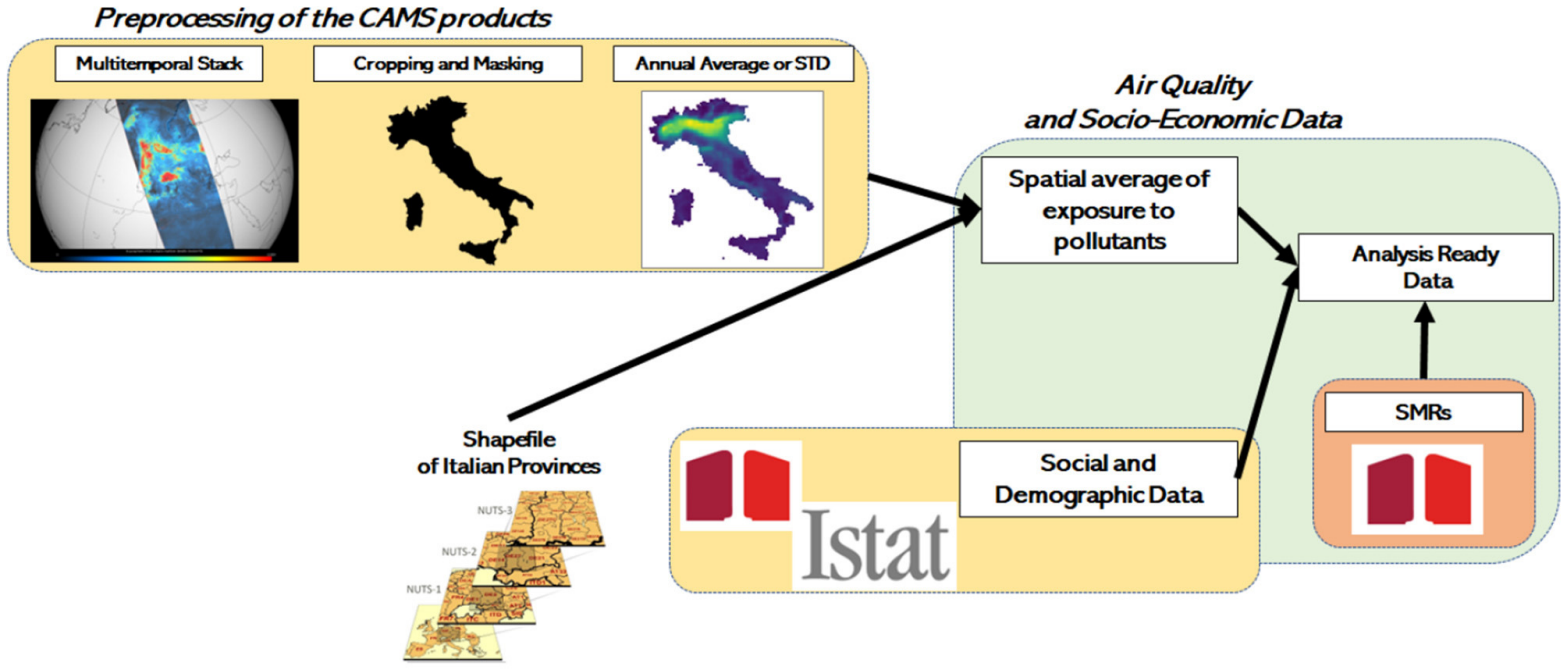
Data preparation workflow.

In this work, we mainly exploited pollutants' concentration from the Copernicus Air Monitoring Service (CAMS). An outline of the data preparation workflow is presented in Figure 1. In order to get an analysis-ready data table we firstly collected daily air quality maps from the Copernicus Data Store for the year 2019.^2^

### 2.1 Input data preparation

The pollution data of year 2019 has been downloaded from Copernicus Atmosphere Monitoring Service (CAMS). It is a European Union program that provides comprehensive information on air quality and the Earth's atmosphere.

It aims to improve air quality forecasts and support decision-making related to air quality management and environmental policy-making in Europe and around the world. CAMS uses a wide range of independent monitoring and modeling systems to collect and analyze data on atmospheric composition, air quality, and weather patterns. CAMS provides annual air quality reanalyzes for Europe based on both unvalidated and validated observations.^3^ Since the downloaded data covered a larger area than that of interest, only pollution data of the Italian peninsula were extracted using the Python library GeoPandas.^4^ Then, for each selected pollutant, both annual average and standard deviation were computed. The polluting substances considered were: carbon monoxide (CO), nitrogen monoxide (NO), nitrogen dioxide (NO_2_), ozone (O_3_), particulate matter 10 (pm10), particulate matter 2.5 (pm2.5) and sulfur dioxide (SO_2_). Pollutant values are the result of an ensemble median of 11 state-of-the-art numerical air quality models developed in Europe: CHIMERE from INERIS (France) (13), EMEP from MET Norway (Norway) (14), EURAD-IM from Jülich IEK (Germany) (15), LOTOS-EUROS from KNMI and TNO (Netherlands) (16), MATCH from SMHI (Sweden) (17), MOCAGE from METEO-FRANCE (France) (18), SILAM from FMI (Finland) (19), DEHM from AARHUS UNIVERSITY (Denmark) (20), GEM-AQ from IEP-NRI (Poland) (21), MONARCH from BSC (Spain) (22) and MINNI from ENEA (Italy) (23). The yearly ensemble reanalyzes are available with a time resolution of 1 h from step 1st January to the 31st of December, while the horizontal resolution of ensemble reanalyzes is on a 0.1° × 0.1° regular latitude-longitude grid. The analysis was conducted using 2019 data since pollution data available on the CAMS platform starts from April 2018. Additionally, years following 2019 were not considered due to the COVID epidemic that began in the early months of 2020, a disease that increased deaths from respiratory illnesses. The average values and standard deviations of pollutants for the provinces of Northern, Central, and Southern Italy are reported in Table 1. Subsequently, in order to get a structured data table to be added to socio-demographic descriptors, a spatial average of these intermediary maps have been computed within the boundaries of each Italian province. Other pollution-related and socio-economic variables were considered to enrich the data set, in particular: cultivated areas, urban areas, benzene, temperature, N fertilizer, P4010 fertilizer, microelement fertilizer, organic fertilizer, bed number, which represents the number of hospital beds available, lifetime, income, life quality, instruction, vehicles total, urban traffic, photovoltaic panel, green urban, electric consumption, noise and wastes (24).

**Table 1:** Mean and standard deviation values of air pollution data by province and pollutant.

**Pollutants**	**Northern Italy**	**Center Italy**	**Southern Italy**
Pm2.5$\left(\frac{\mu g}{m^{3}}\right)$	15.55 ± 5.42	10.94 ± 1.11	11.37 ± 2.15
pm10 $\left(\frac{\mu g}{m^{3}}\right)$	19.09 ± 6.03	15.56 ± 1.37	16.33 ± 2.29
CO$\left(\frac{\mu g}{m^{3}}\right)$	194.04 ± 45.17	156.58 ± 9.24	150.85 ± 20.63
NO$\left(\frac{\mu g}{m^{3}}\right)$	1.23 ± 1.21	0.34 ± 0.16	0.33 ± 0.3
NO_2_$\left(\frac{\mu g}{m^{3}}\right)$	13.15 ± 6.49	7.32 ± 2.55	6.17 ± 3.5
SO^2^$\left(\frac{\mu g}{m^{3}}\right)$	1.56 ± 0.66	1.01 ± 0.27	1.33 ± 0.51
O^3^$\left(\frac{\mu g}{m^{3}}\right)$	58.88 ± 7.79	67.82 ± 5.88	69.63 ± 7.29

The complete list of the selected descriptors is reported in Table 2.

**Table 2:** Overview of the variables selected for this study.

**Data**	**Type**	**Source**	**Value**	**Coding**
Pollutant	Time series $\left(\frac{\mu g}{m^{3}}\right)$	CAMS	Mean $\left(\frac{\mu g}{m^{3}}\right)$, Standard deviation $\left(\frac{\mu g}{m^{3}}\right)$	mean pm2.5, std pm2.5, mean pm10, std pm10, mean CO, std CO, mean NO, std NO, mean NO_2_, std NO_2_, mean O_3_, std O_3_, mean SO_2_, std SO2
Anthropogenic		ISTAT	Mean	N fertilizer, P410 fertilizer, Microelement fertilizer, Organic fertilizer, bed number, life time, income, life quality, instruction, Vehicles total, urban traffic, Electric Consumption, noise, wastes
Environment		ISTAT	Mean	cultivated areas, urban areas, benzene, temperature, photovoltaic panel, green urban

### 2.2 Output data preparation: the indirect standardization

**Figure 2:**
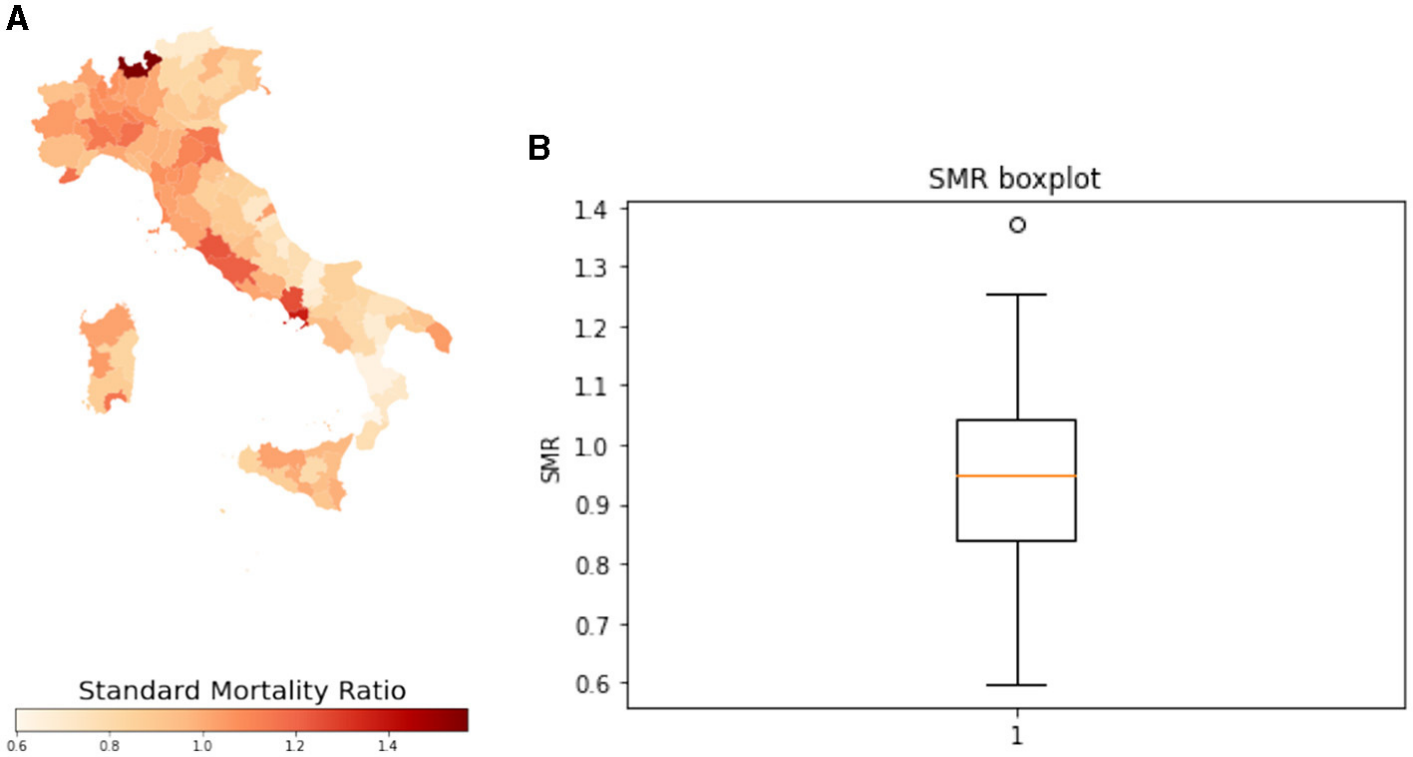
**(A)** Standardized mortality ratio distribution in 2019 within Italian provinces. **(B)** Boxplot of standardized mortality ratio for cancer of the respiratory system of the Italian provinces in 2019.

Indirect standardization is a statistical method used to compare the rates of events or conditions in two or more populations while controlling for differences in population characteristics such as age, sex or socioeconomic status. This method calculates expected rates for each population by applying the distribution of a particular population characteristic (for example age) to a reference population with known rates (25). The observed rates of a particular outcome in each population are then compared to the expected rates, which have been adjusted for any differences in population characteristics. In this work we computed the standard mortality ratio (SMR), the ratio between the deaths observed in a territory and those expected in the same. The expected deaths were calculated by applying the corresponding specific mortality ratios of the population assumed as standard (the national one in this case) to the average annual population by age classes of each territorial unit. The average standardized mortality ratio (SMR for cancer of the respiratory system) distribution within Italian provinces is shown in Figure 2A. Moreover, the province of Sondrio was removed from our analysis since it appears to be an outlier in the SMR distribution, as shown in the Figure 2B.

## 3 Methods

**Figure 3:**
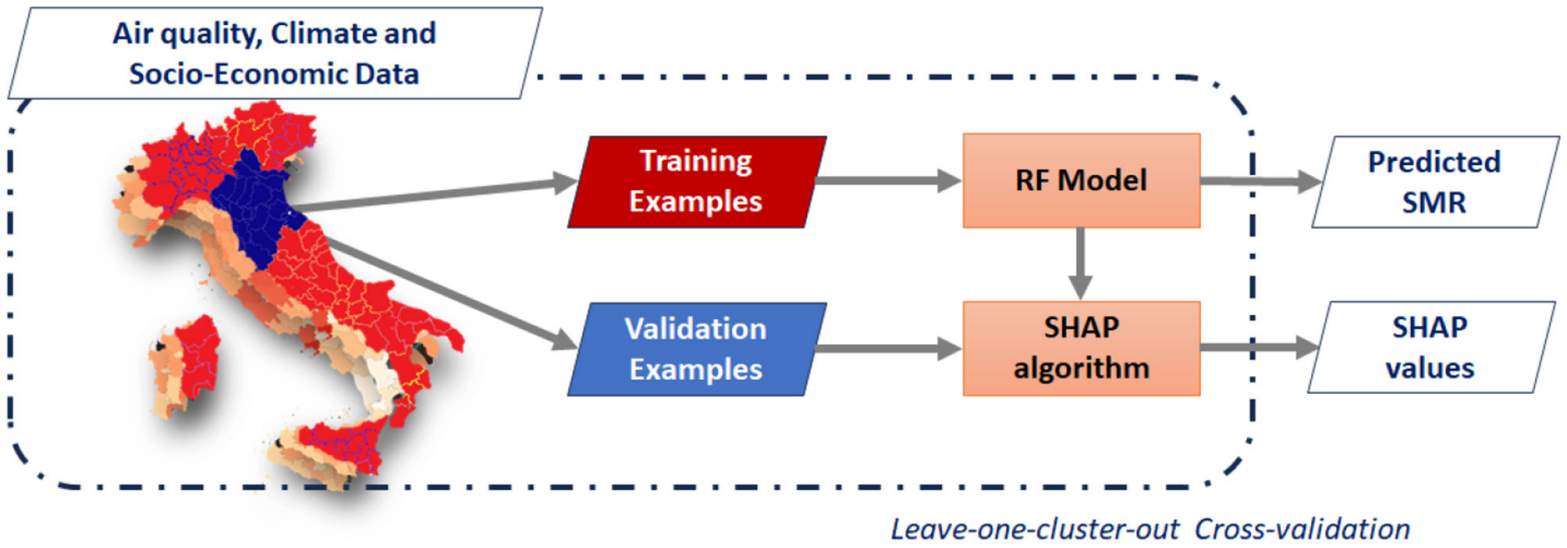
Methodological overview: climate, annual average and standard deviation pollution concentrations and socio-economic variables are used to model the standard mortality ratios (SMRs) in the Italian provinces due to cancer of the respiratory system. Then, the SHAP algorithm is used to compute the contribution of each input variable to the predicted SMRs.

The main goal of this work is to find through an XAI algorithm, which air pollutants and/or socio-economic index contribute most to mortality from lung, trachea and bronchial cancer in Italian provinces. A cross-validation framework has been implemented to train a Random Forest regressor (RF) (26) of the standard mortality ratio for respiratory cancer in Italian provinces; then, by using SHapley Additive exPlanations (27), a method based on cooperative game theory and used, we performed an explain ability analysis to increase transparency and interpretability of machine learning model. The Figure 3 shows a schematic overview of the methods adopted in the present work.

### 3.1 Random forest regressor of standard mortality ratio

Random Forest (RF) operates as an ensemble learning classifier rooted in the concept of classification trees. RF essentially creates a collection of classification trees, wherein each tree is trained on a bootstrapped sample from the available data. To prevent biased estimations, one-third of the available examples are excluded and utilized for the out-of-bag error estimation.

In the process of tree growth, the ideal split at each node relies on a random selection of M/3 descriptors where M represents the total available descriptors. It has been shown that the classification error is influenced by two primary factors: the interdependence among trees in the forest and the individual predictive power of each tree. Managing these factors involves adjusting the number of trees in the forest and the quantity of features sampled per split. The accuracy of RF models substantially depends on two parameters, the number of sampled features f and the number of the forest trees T.

In this study, RF was implemented using the random Forest routine from the scikit-learn package (v 1.2.1) (28) with its default configuration.

### 3.2 Hierarchical spatial clustering and model performance assessment

In order to avoid information leakage between spatially dependent observations, we preliminary found a partition of clusters of adjacent provinces to keep apart during the training and validation steps.

The spatial clusters of provinces adopted in the cross-validation scheme were found through a combined use of a hierarchical clustering algorithm applied to the matrix of the euclidean distances between provinces, and the semivariogram plot of the SMRs. A semivariogram plot is a tool used in geostatistics to assess the spatial dependence of a variable. It is usually plotted on a graph where the x-axis corresponds to the distance between a pair of selected locations (also known as spatial lag), while the y-axis corresponds to the average squared difference of the variable values computed for those pairs of location within a given distance interval. In this work, we estimated the spatial range of the semivariogram, i.e., the distance after which the observations are supposed to be no longer correlated, using a discretionary approach by a visual identification of the plateau (i.e., when no further increase in variance is observed).

**Figure 4:**
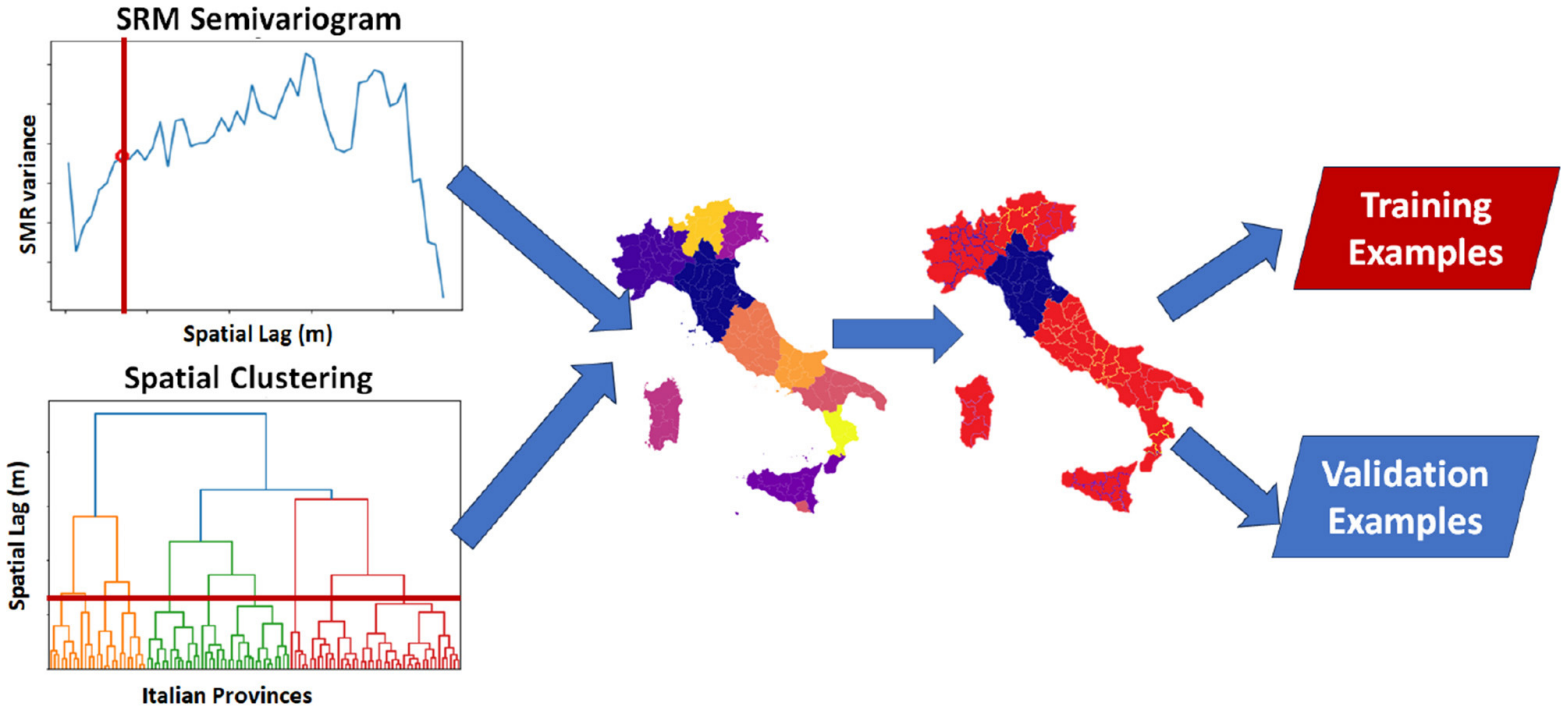
The hierarchical clustering algorithm used in combination with the semivariogram plot of the SMR rates delineated 10 spatially-contiguous community that alleviated the spatial data leakage that might occur in epidemiological studies.

Establishing the range of the empirical semivariogram of the SMRs allowed us to delineate ten clusters after thresholding the dendrogram obtained by the hierarchical clustering of the geographical distances of the Italian provinces (Figure 4).

We utilized a leave-one-cluster-out cross-validation approach to mitigate spatial data bias when assessing the regression performance of an ensemble Random Forest regressor. This validation scheme was implemented to prevent overestimation of performance. In fact, spatial autocorrelation in two adjacent provinces, one selected in the training set and the other in validation, may lead to overoptimistic results.

Finally, we evaluated the performance of our machine learning model, by computing the coefficient of determination *r*^2^ (Equation 1) and the mean absolute error *MAE* (Equation 2), whose definition is provided in the following:

Coefficient of determination:


(1) $$r^{2}=1-\frac{\sum_{i=1}^{n}{\left(y_{i}-{\hat{y}}_{i}\right)}^{2}}{\sum_{i=1}^{n}{\left(y_{i}-\bar{y}\right)}^{2}}$$

Mean absolute error:


(2) $$\begin{array}{l} MAE=\frac{1}{n}\overset{n}{\underset{i-1}{\sum }}|\left(y_{i}-{ŷ}_{i}\right)| \end{array}$$

where ${\hat{\text{y}}}_{\text{i}}$ are the predicted values, $\bar{\text{y}}$ is their average, while *y*_*i*_ are the observed values of the SMR in the validation set.

### 3.3 Features explain ability

Epidemiological studies are crucial in understanding and controlling the spread of diseases, but often require large sets of data that are difficult for humans to analyze efficiently. Artificial intelligence (AI) has emerged as a useful tool in epidemiological studies, with promising applications in predicting disease outbreaks, identifying risk factors, and developing targeted interventions. However, as AI becomes more prevalent in the field, there is a growing concern about its lack of transparency and explain ability, which can limit its utility and undermine the trust in its results. Explainable artificial intelligence (XAI) can address these concerns by providing interpretable models, transparent decision-making processes, and clear explanations of the AI's predictions and recommendations. In this work, we used SHapley Additive exPlanations (SHAP) method, a XAI algorithm borrowed from game theory (29, 30). The SHapley Additive exPlanations (SHAP) method is a model-agnostic approach to interpret the output of any machine learning model. It provides a unified framework for interpreting the predictions of any model by assigning a feature importance value to each input feature (Equation 3).

SHAP method values how a feature affects the performance of the model on the validation set by including and removing it from the model:


(3) $$\begin{array}{l} \Phi_{j}\left(x\right)=\underset{F\subseteq S-\left\{j\right\}}{\sum }\frac{|F|!\left(|S|-|F|-1\right)!}{|S|!}\left[f_{x}\left(F\cup j\right)-f_{x}\left(F\right)\right] \end{array}$$

where x is an instance, the sum is over all the subsets S of features which include the feature j, $\frac{|F|!\left(|S|-|F|-1\right)!}{|S|!}$ is a weight parameter that multiplies all of the permutations of S! by the potential permutations of the remaining class that doesn't belong to S, while *fx*(*F* ∪ *j*) and *fx*(*F*) denote respectively the regression score obtained by including and non-including feature j.

## 4 Results

The goal of this work was to evaluate, through explainable machine learning models, whether and to what extent air pollutants and socio-economic descriptors are associated with mortality due to respiratory cancer.

**Figure 5:**
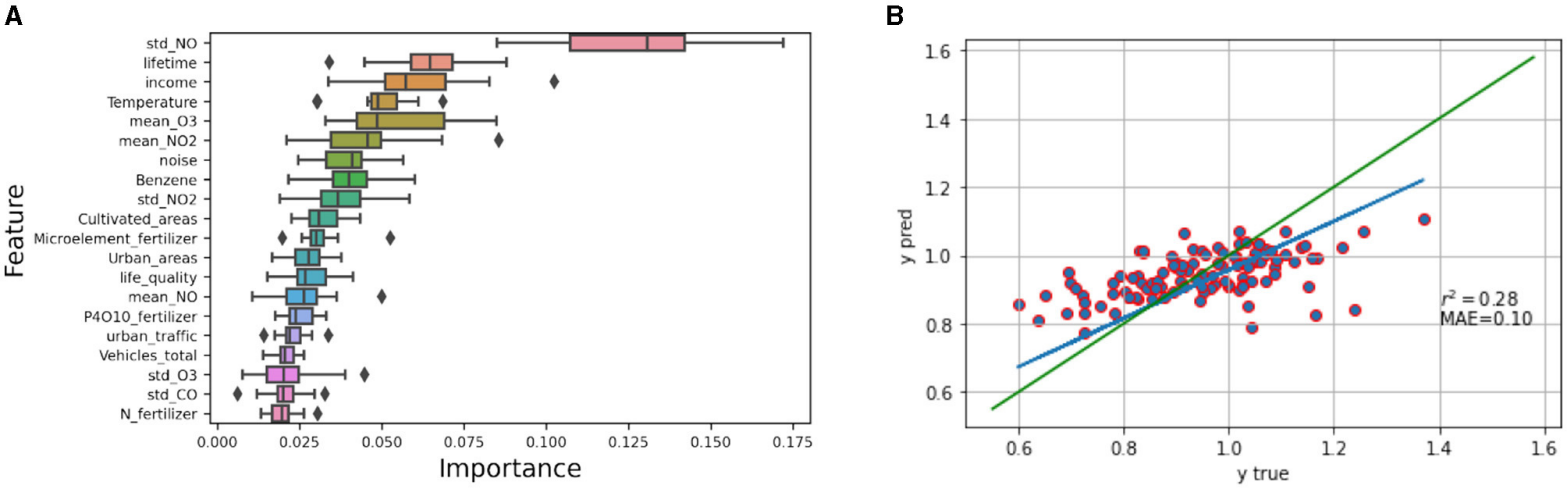
**(A)** Random forest features importance. **(B)** Scatter plot of predicted Standard Mortality Ratio (y pred) vs. true Standard Mortality Ratio (y true). The blue line represents the best fit for the plotted points, whereas the green one represents where the points would fall if all predicted values perfectly matched the observed ones.

Following the procedures described in the methods section, we delineated 10 clusters when using the spatial range value from the semivariogram plot to split the dendrogram of the geographical distances between the Italian provinces. Then, a leave-one cluster-out validation scheme was adopted in order to get a robust assessment of the model performance. We quantitatively assessed the model performance in terms of the average metrics obtained on the validation set; our model achieved an *r*2 value of 0.28 and a mean absolute error (*M AE*) value of 0.10. Moreover, the importance of all available variables included in the analysis was computed by exploiting the permutation feature importance algorithm and plotted in Figure 5A. Finally, a scatter plot displaying the mutual agreement between the actual and predicted Standard Mortality Ratios is shown in Figure 5B. The provinces characterized by the highest SMR in the 2019 are located in the right-most part of the scatterplot; these are the provinces of Napoli, Caserta, Viterbo, Roma, Piacenza, Imperia, Ravenna, Cagliari, Alessandria and Ferrara.

**Figure 6:**
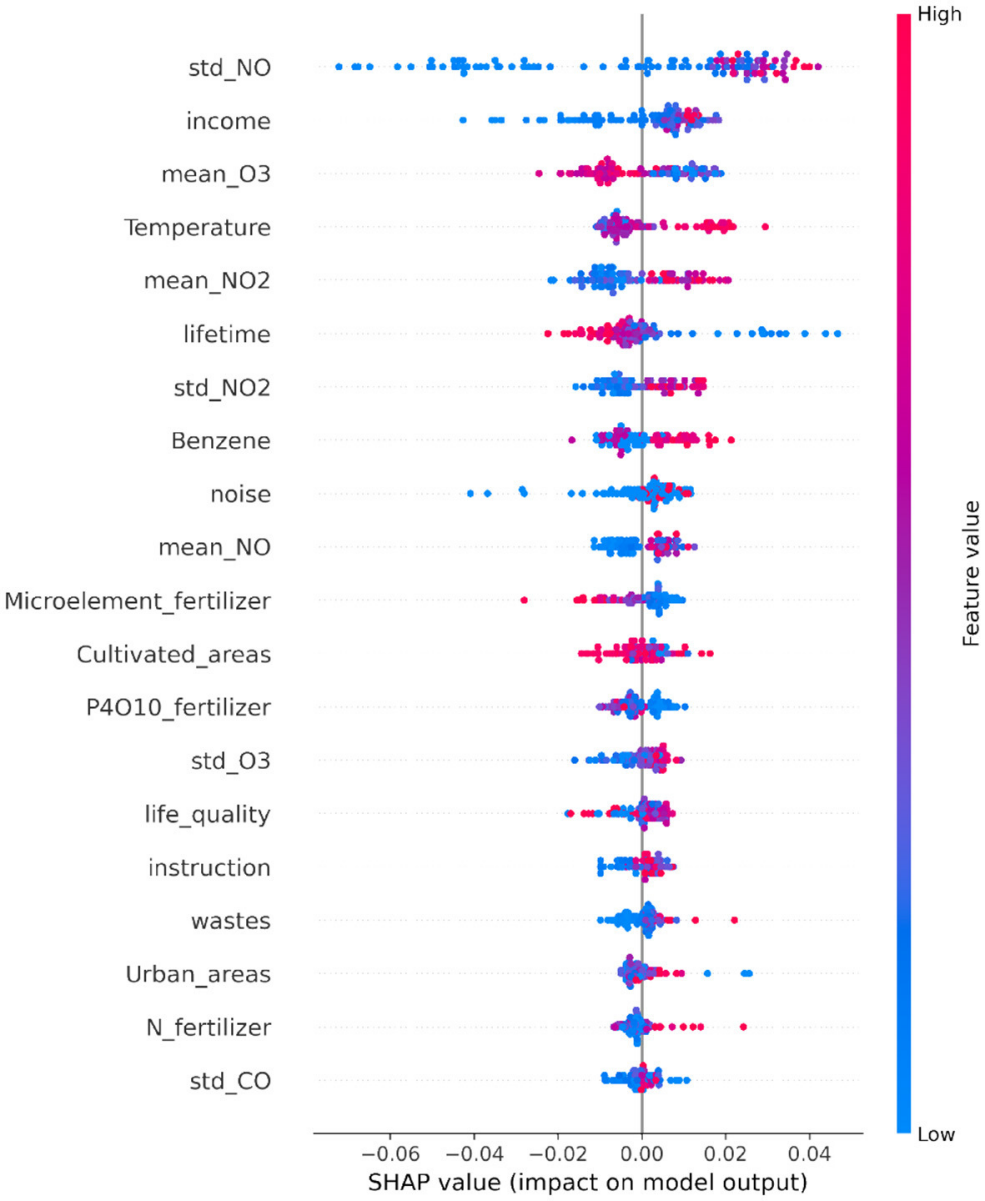
SHAP summary plot illustrating the SHAP values for each feature. Each point on the plot represents a Shapley value of a provinces, with the y-axis indicating the corresponding feature and the x-axis representing the Shapley value itself. The color gradient reflects the feature value, ranging from low to high. The features are ordered based on their mean importance, with more important features positioned toward the top.

Figure 6 shows the most important features for regression according to the SHAP algorithm. This summary plot offers an overview of the varying degrees of influence of every feature on the model's predictions, thereby enabling a better grasp of the comprehensive significance and effect of distinct features in the analysis.

According to the SHAP summary plot, the top 5 most important variables include three related to pollution (std NO, mean O_3_, mean NO_2_), one associated with climate (temperature), and another tied to social factors (income).

## 5 Discussion

The performances obtained in terms of r2 and MAE are respectively 0.28 and 0.10. This result shows that environmental pollution is associated to the considered type of cancer. From the analysis conducted, it emerges that there is an association between certain pollutants and the incidence of respiratory system cancer. This information should be included alongside other risk factors in studies investigating risk factors for individual subjects, including personalized ones (31, 32).

The spatial auto-correlation analysis, performed by using the variogram of the standard mortality ratio, shown ten clusters of provinces. The cross validation performed by using these clusters, obtained an r2 lower than one with a random cross-validation, by showing a spatial bias that overestimated the random forest regressor performance.

XAI estimated which are the most important global and local features in predicting SMR. In particular, for the present case, the main role played by the standard deviation of NO and mean O_3_ were revealed. This result is consistent with the literature, as it is known that a greater exposure to NO_2_ is correlated with a greater risk of developing lung cancer (33). According to our study, a low average O_3_ concentration is linked to a greater SMR for tumors of the respiratory system. Such a spurious association have been observed before in previous works, including Dutton et al. (34) and Travaglio et al. (35), and we believe that a plausible interpretation is that increased ozone exposure is acting a as proxy of residing in rural areas (36, 37). Moreover, it is also known that the concentration of O_3_ is anti-correlated to the concentration of NO_2_ (36–42). This spatial pattern between rural areas and O_3_ concentration is rather general, as O_3_ in troposphere is a secondary pollutant that is produced after NO_2_ reacting with UV light (40, 43). We also believe that the observed negative correlation between NO_2_ and O_3_ can be largely attributable to this causal link.

Concerning the socio-demographic descriptors, income and lifetime were the most important. Higher income appears to be positively associated with SMR, implying greater cancer exposure among wealthier individuals. Such association is in stark contrast with the part of the current literature supporting the evidence of how social inequalities may increase exposure to poor air quality and vulnerability to respiratory diseases (34, 44, 45). Richardson et al. (44) claimed evidence of income-related inequalities in exposure to pollutants. Studies conducted to individual-level demographic data that accounts social exclusion and ethnicity (34, 45), evidenced that individual belonging to ethnic minorities are disproportionately exposed to poor air quality. However, this pattern has not been observed in Italy. Germani et al. (46), provided an empirical analysis conducted on the Italian provinces (NUTS3) claiming that air pollution increases with the average income per administrative unit. Since our study relies on provinces, coarse air quality products and no individual-level data is included to account for social and gender, we believe that the average income per province may act as a proxy for both human activities and may not point to social exclusion due to the coarse resolution of the proposed study.

Lifetime duration shows a consistent trend with expected associations: shorter lifespans correspond to higher mortality rates from respiratory diseases. We might speculate that respiratory diseases significantly impact life expectancy in Italy, particularly in certain provinces, potentially influencing the overall health of the population.

The presented study has limitations that we aim to overcome in future research. In particular, the database considered here examined the remote sensing observations of 2019 of exposure to air pollutants. An extension of the time range of our study would guarantee greater robustness to the analyses. Moreover, another limitation of this study is the failure to account for the population density differences within the provinces, as it varies between small urban centers and large cities, implying uniform exposure to air pollutants for the entire population of a province.

## 6 Conclusion

The presented analysis reveals a correlation between specific pollutants and socio-economic indices and the occurrence of respiratory system cancer. These finding could still offer valuable insights for further epidemiological studies as our results may suggest which variables to gather to perform analyses on individual level dataset that could lead to stronger and more conclusive results. Moreover, this study opens up future prospects for similar research on other types of cancer related to environmental pollution, as well as other types of diseases such as neurodegenerative ones.

## Data availability statement

The original contributions presented in the study are included in the article/supplementary material, further inquiries can be directed to the corresponding authors.

## Author contributions

DR: Writing—review & editing, Writing—original draft, Visualization, Validation, Software, Methodology, Investigation, Formal analysis, Data curation, Conceptualization. PN: Writing—review & editing, Validation, Writing—original draft, Methodology, Investigation, Conceptualization. RC: Writing—review & editing, Writing—original draft, Validation, Methodology. NA: Writing—review & editing. AM: Writing—review & editing, Validation, Methodology. RB: Writing—review & editing, Supervision, Methodology, Funding acquisition. ST: Writing—review & editing, Writing—original draft, Visualization, Supervision, Project administration, Methodology, Funding acquisition, Conceptualization.

## Footnotes

^1^Available online at: https://www.istat.it/it/archivio/222527.

^2^Available online at: https://atmosphere.copernicus.eu/.

^3^Available online at: https://ads.atmosphere.copernicus.eu/#!/search?text=andtype=datasetandkeywords=((%20%22Product%20type:%20Reanalysis%22%20)%20AND%20(%20%22Variable%20domain:%20Atmosphere%20(composition)%22%20)%20AND%20(%20%22Spatial%20coverage:%20Europe%22%20)%20AND%20(%20%22Temporal%20coverage:%20Past%~22%20)).

^4^Available online at: https://geopandas.org/en/stable/.
